# Paralysis and Necrotic Wound Infection Resulting From Monocled Cobra Envenomation

**DOI:** 10.7759/cureus.72875

**Published:** 2024-11-02

**Authors:** Danielle A Sultan, George Angelakakis, Matthew C Braun, John D DelBianco, Kenneth D Katz

**Affiliations:** 1 Department of Emergency and Hospital Medicine, Lehigh Valley Health Network/University of South Florida Morsani College of Medicine, Allentown, USA; 2 Department of Family Medicine, Northern Light-Eastern Maine Medical Center, Bangor, USA; 3 Department of Emergency and Hospital Medicine, Division of Medical Toxicology, Lehigh Valley Health Network/University of South Florida Morsani College of Medicine, Allentown, USA

**Keywords:** antivenom, envenomation, monocled cobra, non-native snake bite, toxinologist

## Abstract

Non-native snake envenomations can be difficult to manage because of challenges obtaining appropriate antivenom and unfamiliarity with the expected clinical effects. This case report describes a 37-year-old man who was envenomated by his pet monocled cobra (*Naja kaouthia*). He experienced respiratory failure, requiring intubation and mechanical ventilation. Thai king cobra antivenom, recommended by a toxinologist consultant, was obtained from the nearby zoo and administered. The patient was extubated on hospital day (HD) 2. He returned to the hospital two days after initial discharge with worsening erythema, swelling, and purulent discharge from the bite wound. He was treated in the emergency department (ED) and started on intravenous vancomycin and cefepime. The wound culture report found *Morganella morganii* and *Enterococcus faecalis*. The patient transitioned to oral antibiotics at discharge on HD 6. The wound developed an eschar, so he underwent debridement, fasciotomy and skin grafting on an outpatient basis. Thirty-six days post-envenomation, he achieved full functional recovery. This case demonstrates some of the challenges inherent to the management of envenomations by non-native snakes. Medical toxicologists and poison control centers can help to find appropriate antivenom and guide treatment.

## Introduction

An estimated 7,000 to 8,000 venomous snake bites occur annually in the United States (US) [1-3]. Although most envenomizations occur from snakes endemic to the US such as rattlesnakes, copperheads, cottonmouths, and coral snakes, there were 258 confirmed non-native snake bites in the US from 2005 through 2011 [1]. Elapids accounted for 100 envenomations, vipers for 118, and the remaining were unknown or unable to be classified [1]. Fifty-eight of the 100 elapids were true cobras (genus *Naja*), and another six were King Cobras (genus *Ophiophagus*) [1-3]. Monocled cobras (*Naja kaouthia*) are native to Africa and Asia but may be seen in zoos or owned privately by individuals in the US [4]. There has been an increase in exotic snake envenomation in private residences [1,4] with approximately 70% of non-native bites occurring in this location [1,5]. Severe envenomation by a monocled cobra requiring emergent airway control, antivenom administration, and surgical wound debridement is described in this case report.

This case was presented, in part, at the American College of Medical Toxicology 2024 Annual Scientific Meeting & Symposia in Washington, DC (April 10-14, 2024) and the Pennsylvania College of Emergency Physicians 2024 Scientific Assembly in Pocono Manor, PA (May 2-4, 2024).

## Case presentation

A 37-year-old male, with a past medical history of bipolar disorder, generalized anxiety disorder, and deafness, presented to the hospital after sustaining a right forearm bite by his pet monocled cobra. Communication was provided through American Sign Language interpretation. After sustaining the bite, he began to complain of dizziness and vomiting. It is unclear what time the bite occurred. Within nine minutes of emergency medical services (EMS) contact, the patient had rapidly declining mental and respiratory status requiring bag-valve-mask (BVM) ventilation for respiratory arrest within eleven minutes. Upon arrival at the emergency department (ED), he was being ventilated by BVM, and his Glasgow Coma Scale was 3. He was emergently endotracheally intubated due to acute respiratory failure. His initial vital signs following intubation were temperature 36.6°C, heart rate 95 beats per minute, respiratory rate 26 breaths per minute, blood pressure 210/106, and oxygen saturation 95% on 15 liters per minute of supplemental oxygen by BVM. Point-of-care glucose was 231 mg/dL. The patient’s initial diagnostic studies were remarkable only for leukocytosis (23,200/cmm [4,000-10,500/cmm]), hypokalemia (3.4 mmol/L [3.5-5.2 mmol/L]), and lactemia (4 mmol/L [0.5-2.1 mmol/L]). The remainder of his laboratory testing, including electrolytes, renal function, and liver enzymes, were normal. The patient was admitted to our institution’s regional level one trauma and burn center for further management.

The medical toxicology service was consulted. After an immediate and extensive search for appropriate antivenom, a stock of equine-derived King Cobra antivenom was identified at a large regional zoo located approximately 60 miles from the hospital. The antivenom was transported by helicopter to the hospital and administered intravenously (100mL) approximately six hours after the patient's arrival. The patient demonstrated spontaneous head movements within six hours of antivenom administration. He was extubated on hospital day (HD) 2.

**Figure 1:**
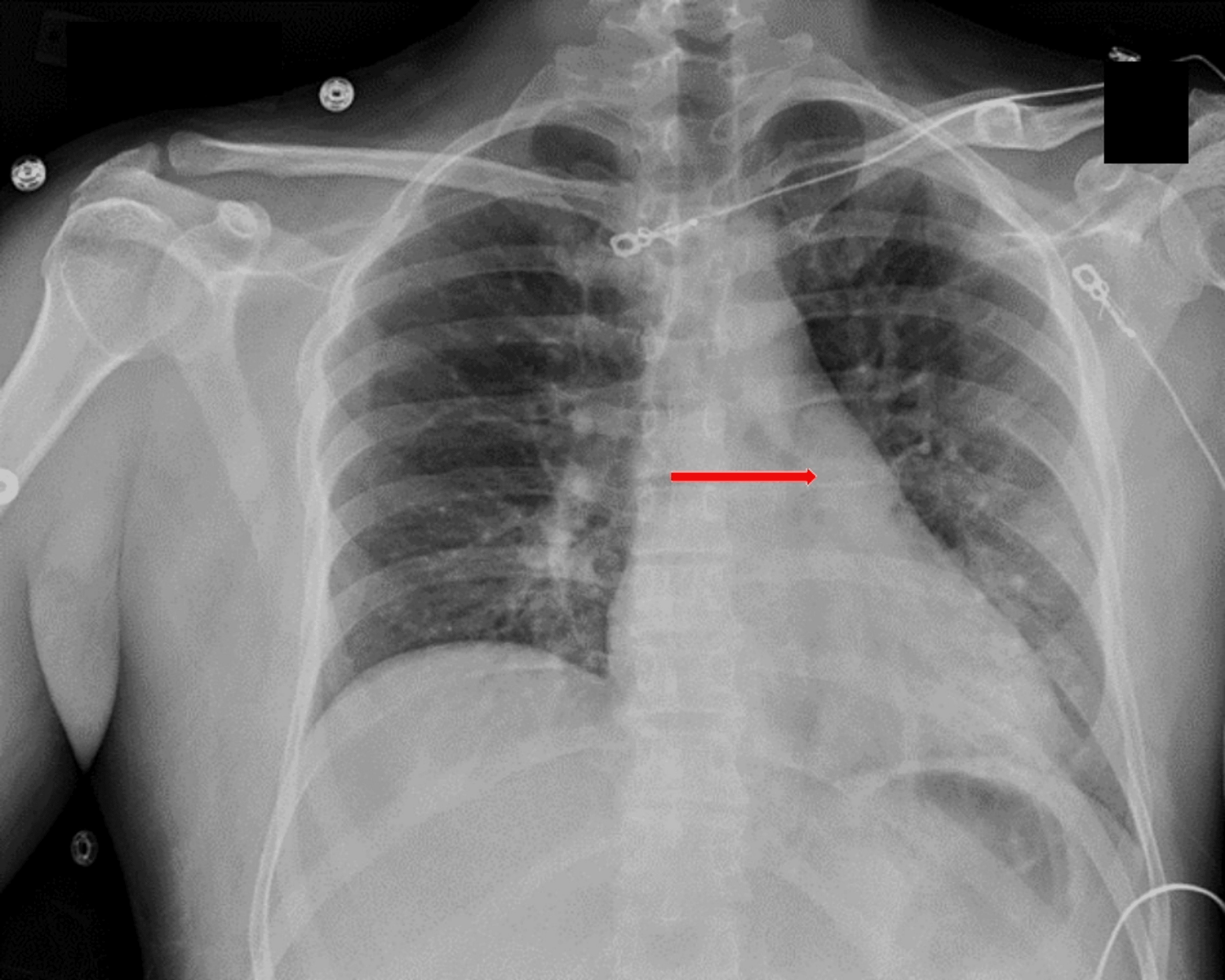
Chest radiograph with arrow showing opacification in the left lower lobe

Intravenous fluids were administered for elevated creatinine phosphokinase, which peaked at 2,564 U/L [<351 U/L]. His high sensitivity troponin rose to a peak of 144 ng/L [<80 ng/L] at hour three on HD 1, which improved without intervention. Due to the patient’s elevated troponin, a transthoracic echocardiogram was performed on HD 2 with no abnormal findings. The bite wound was painful, erythematous, and warm. Antibiotics were considered but withheld because his fever and leukocytosis were thought to be secondary to aspiration pneumonitis, as serial chest radiographs showed the development of opacifications in the left lower lobe (Figure 1). The extremity erythema, swelling, and pain improved without antibiotic treatment. His tetanus vaccination was updated. He was discharged in normal neurologic condition on HD 3 without antibiotics. There was a small wound at the bite site without drainage.

**Figure 2:**
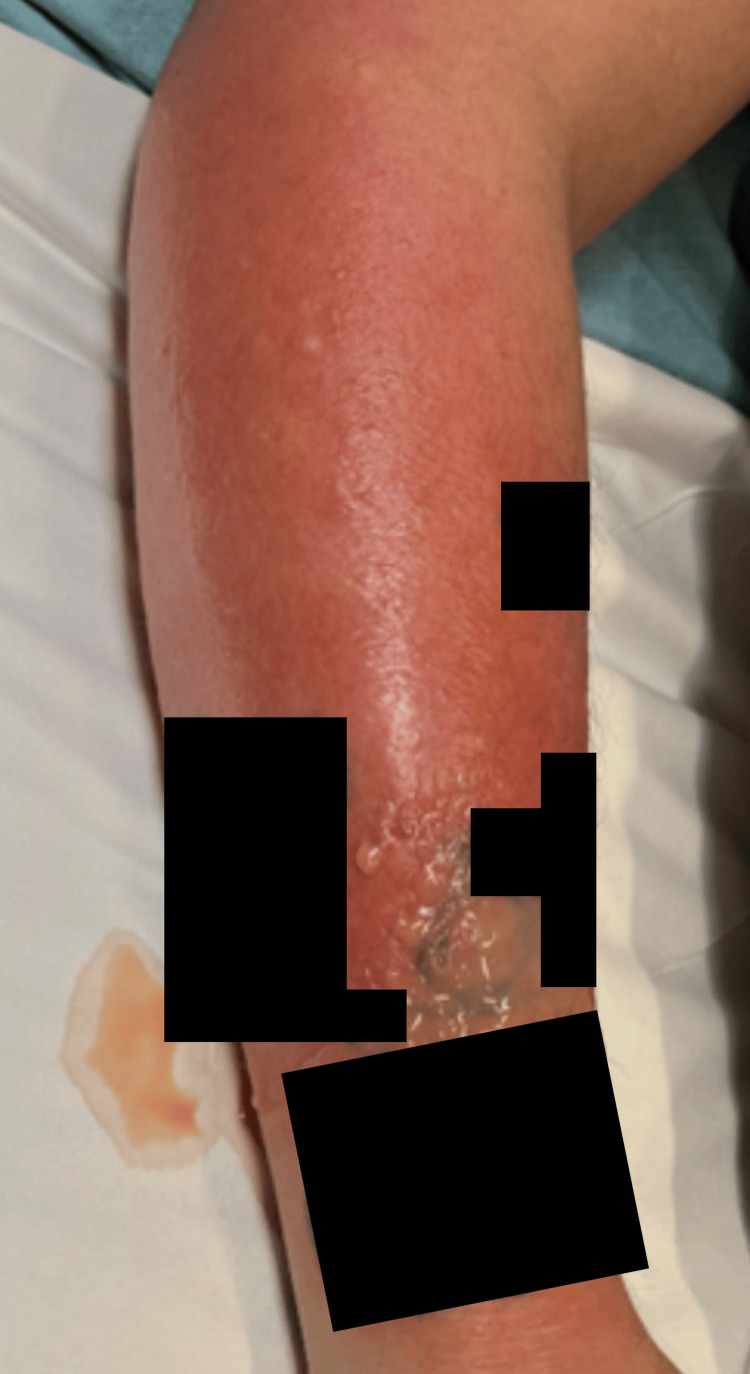
Photo of patient’s arm five days post-envenomation

Two days after discharge and five days post-envenomation, the patient returned to the ED with purulent drainage from the bite wound and surrounding erythema and swelling (Figure 2). His white blood cell count was 15,200/cmm [4,000-10,500/cmm]. He was treated in the ED with intravenous vancomycin and cefepime and admitted. The wound developed an eschar, prompting surgical evaluation and a plan for operative debridement. A wound culture grew *Morganella morganii *and *Enterococcus faecalis*. Surgery was ultimately deferred to the outpatient setting per the patient’s request. He was discharged on HD 6 of his second hospitalization and prescribed a 10-day regimen of oral amoxicillin-clavulanic acid, ciprofloxacin, and topical bacitracin. He did not adhere to the prescribed regimen, taking only one of the antibiotics but was unsure which one he was taking. Fifteen days post-envenomation, he underwent outpatient elective debridement, fasciotomy and skin grafting. During a follow-up visit on day 36 post-envenomation, he achieved full functional recovery. The patient’s significant other reported that she gave the snake to an animal rescue organization.

## Discussion

This case highlights significant challenges inherent to the assessment and management of a patient envenomated by a non-native snake. Medical toxicologists and poison control centers are invaluable to both assisting treating physicians in the procurement of antivenom as well as providing specialized medical care. Exotic species' antivenom can be stored at regional zoos which are urged to maintain antivenom specific to the venomous animals in their care. The Association of Zoos and Aquariums, the accrediting body for many zoos in the US and internationally, maintains an online database that lists various antivenoms stocked by its member institutions and species for which these antivenoms are expected to be effective [6]. Procuring exotic antivenoms in a timely manner outside of the US can also prove challenging. France and the Netherlands have antivenom banks that stock critical antivenom for native and non-native snake envenomations [1]. In this case, King Cobra antivenom was recommended by a medical toxinologist consultant. Successful treatment of monocled cobra envenomation with King Cobra antivenom has been previously reported [7].

Transporting the antivenom from the zoo to the hospital presents additional challenges, since the antivenom may need to be transported a long distance and must remain temperature controlled. In this case, a medical flight crew retrieved the Thai King Cobra antivenom from the zoo in a refrigerated container and transported it by helicopter to the hospital pharmacy, where pharmacists and toxicologists were waiting to reconstitute and administer it. Planning and protocol development involving regional poison centers, hospitals, Emergency Medical Services (EMS), and local zoos may help to facilitate the coordinated response to a non-native envenomation [8].

Understanding the potential complications of envenomation is paramount to providing appropriate resuscitation of the critically ill patient. Cobra venom contains multiple toxins which cause neurotoxicity, cardiotoxicity, and local tissue damage [4, 5, 7, 9-12]. The major toxic proteins found in venom are ⍺-neurotoxins, phospholipase A2, cysteine-rich secretory protein, snake venom metalloprotease, and cytotoxins [13]. The clinical consequences of envenomation can vary among members of the same species, depending on the snake’s geographic location, diet, and species, which can alter the chemical composition of the venom.

Paralysis is mediated by the actions of ⍺-neurotoxins via competitive inhibition of postsynaptic nicotinic receptors in muscle tissue as well as beta-neurotoxins that damage presynaptic nerve terminals, thereby depleting neurotransmitters [9,14,15]. Paralysis is typically descending and may begin with early clinical signs such as ptosis and ophthalmalgia, followed by limb weakness and respiratory failure from paralysis of the intercostal and diaphragm muscles [9,15].

The patient described in this case had mildly elevated high-sensitivity troponin which improved spontaneously without echocardiogram abnormalities. The mechanism for cardiotoxicity is still not well understood but reported venom effects include chest pain, troponin elevations, electrocardiogram changes, hypertension, myocardial infarction, and dysrhythmias. The troponinemia may have represented a direct effect of cardiotoxic proteases in the snake venom disturbing cardiac membrane proteins and inducing myocardial ischemia. Another possibility is cardiac demand ischemia resulting from respiratory arrest and physiologic stress from intubation. The patient in this case was managed supportively and improved without intervention or sequelae.

This patient was not initially treated with antibiotics for the localized extremity swelling and redness because swelling, erythema, pain, leukocytosis, and fever have all been described to result from snake envenomation [16]. Moreover, fever and leukocytosis may have been related to aspiration pneumonitis or administration of the equine-derived antivenom. The patient also improved clinically at the time of discharge. However, purulent discharge and persistent leukocytosis at his second ED visit prompted the initiation of antibiotics.

Dermatotoxicity is mediated by cytokine disruption of membrane-bound proteins and activation of apoptotic and necrotic cell death pathways [17]. In more severe cases, patients can suffer local wound infection requiring operative debridement and fasciotomy [2,5,10,11,16] with some studies suggesting a dose-dependent correlation with the severity of wound infection [18]. Necrotic wounds are often polymicrobial, with pathogens generally reflective of the cobra’s oral bacterial flora transmitted through direct inoculation [18]. Common pathogens include *Morganella morganii* as well as *Enterococcus faecalis* [2,18,19] and *Pseudomonas sp.* [16].

Leukocytosis has been described after envenomation by a variety of snakes, including the monocled cobra [10,20]. It may be related to enzymes in the venom that incite an inflammatory response and exacerbated by tissue necrosis [20]. The clinical significance of this common laboratory finding is unclear. One study comparing infected to non-infected *Naja atra* bites found a significantly higher neutrophil-lymphocyte ratio in the infected group, but the white blood cell count did not differ significantly between groups [18]. This patient’s peak leukocytosis was noted immediately after envenomation and before the development of infection, which likely represented an inflammatory response to the venom itself. The persistence of leukocytosis days after envenomation may have been multifactorial and related to aspiration pneumonitis, antivenom, tissue necrosis, and the local wound infection.

The role of empiric antibiotic administration remains controversial; however, treatment of overt wound infections from envenomation should target the aforementioned bacteria species [16]. The patient in this case had clinical improvement after treatment with cefepime, amoxicillin-clavulanic acid, ciprofloxacin, and bacitracin for his polymicrobial wound infection. He required skin grafting to make a full recovery. Non-adherence to the prescribed antibiotic regimen also may have contributed to the need for surgery.

## Conclusions

This patient required intubation for respiratory failure after envenomation by a monocled cobra. He was treated with Thai King Cobra antivenom obtained from a local zoo. He developed myocardial injury as well as cellulitis and necrosis at the site of the envenomation which were treated with antibiotics and skin grafting. He eventually made a full functional recovery.

Envenomations by snake species not native to the US, including the monocled cobra, are challenging to manage because of difficulty obtaining antivenom and unfamiliarity with the clinical effects. Clinicians should be aware that monocled cobra envenomation can result in paralysis, cardiotoxicity and dermal necrosis. Consultation with a poison center or medical toxicologist is critical in all cases not only to assist in procuring exotic antivenom but also to provide specialized care to the envenomated patient.

## References

[1] Warrick BJ, Boyer LV, Seifert SA (2014). Non-native (exotic) snake envenomations in the U.S., 2005-2011. Toxins (Basel).

[2] Basse J, Ruha AM, Baumgartner K (2023). Clinical presentations, treatments, and outcomes of non-native snake envenomations in the United States reported in the North American Snakebite Registry. J Med Toxicol.

[3] Seifert SA, Armitage JO, Sanchez EE (2022). Snake envenomation. N Engl J Med.

[4] Miller SW, Osterhoudt KC, Korenoski AS, Patel K, Vaiyapuri S (2020). Exotic snakebites reported to Pennsylvania poison control centers: lessons learned on the demographics, clinical effects, and treatment of these cases. Toxins (Basel).

[5] Fuchs J, Gessner T, Kupferschmidt H, Weiler S (2022). Exotic venomous snakebites in Switzerland reported to the National Poisons Information Centre over 22 years. Swiss Med Wkly.

[6] Seifert SA, Keyler D, Isbister G, McNally J, Martin TG (2006). ACMT Position Statement: Institutions housing venomous animals. J Med Toxicol.

[7] Jagpal PS, Williams HA, Eddleston M (2022). Bites by exotic snakes reported to the UK National Poisons Information Service 2009-2020. Clin Toxicol (Phila).

[8] Othong R, Sheikh S, Alruwaili N, Gorodetsky R, Morgan BW, Lock B, Kazzi ZN (2012). Exotic venomous snakebite drill. Clin Toxicol (Phila).

[9] Tan CH, Bourges A, Tan KY (2021). King Cobra and snakebite envenomation: on the natural history, human-snake relationship and medical importance of Ophiophagus hannah. J Venom Anim Toxins Incl Trop Dis.

[10] Faiz MA, Ahsan MF, Ghose A (2017). Bites by the monocled cobra, Naja kaouthia, in Chittagong Division, Bangladesh: epidemiology, clinical features of envenoming and management of 70 identified cases. Am J Trop Med Hyg.

[11] Wongtongkam N, Wilde H, Sitthi-Amorn C, Ratanabanangkoon K (2005). A study of Thai cobra (Naja kaouthia) bites in Thailand. Mil Med.

[12] Ismail AK (2015). Snakebite and envenomation management in Malaysia. Clinical Toxinology in Asia Pacific and Africa.

[13] Liu CC, Chou YS, Chen CY (2020). Pathogenesis of local necrosis induced by Naja atra venom: assessment of the neutralization ability of Taiwanese freeze-dried neurotoxic antivenom in animal models. PLoS Negl Trop Dis.

[14] Gasanov SE, Dagda RK, Rael ED (2014). Snake venom cytotoxins, phospholipase A(2)s, and Zn(2+)-dependent metalloproteinases: mechanisms of action and pharmacological relevance. J Clin Toxicol.

[15] Ranawaka UK, Lalloo DG, de Silva HJ (2013). Neurotoxicity in snakebite--the limits of our knowledge. PLoS Negl Trop Dis.

[16] Mao YC, Liu PY, Hung DZ, Lai WC, Huang ST, Hung YM, Yang CC (2016). Bacteriology of Naja atra snakebite wound and its implications for antibiotic therapy. Am J Trop Med Hyg.

[17] Kalita B, Utkin YN, Mukherjee AK (2022). Current insights in the mechanisms of cobra venom cytotoxins and their complexes in inducing toxicity: implications in antivenom therapy. Toxins (Basel).

[18] Yeh H, Gao SY, Lin CC (2021). Wound infections from Taiwan cobra (Naja atra) bites: determining bacteriology, antibiotic susceptibility, and the use of antibiotics-a cobra BITE study. Toxins (Basel).

[19] Huang WH, Kao CC, Mao YC (2021). Shewanella algae and Morganella morganii coinfection in cobra-bite wounds: a genomic analysis. Life (Basel).

[20] Zuliani JP, Soares AM, Gutiérrez JM (2020). Polymorphonuclear neutrophil leukocytes in snakebite envenoming. Toxicon.

